# Inbreeding Depression in *Solanum carolinense* (Solanaceae) under Field Conditions and Implications for Mating System Evolution

**DOI:** 10.1371/journal.pone.0028459

**Published:** 2011-12-12

**Authors:** Rupesh R. Kariyat, Sarah R. Scanlon, Mark C. Mescher, Consuelo M. De Moraes, Andrew G. Stephenson

**Affiliations:** 1 Department of Biology, The Pennsylvania State University, University Park, Pennsylvania, United States of America; 2 Department of Entomology, The Pennsylvania State University, University Park, Pennsylvania, United States of America; University of Western Ontario, Canada

## Abstract

The clonal weed *Solanum carolinense* exhibits plasticity in the strength of its self-incompatibility (SI) system and suffers low levels of inbreeding depression (δ) in the greenhouse. We planted one inbred and one outbred plant from each of eight maternal plants in a ring (replicated twice) and monitored clonal growth, herbivory, and reproduction over two years. Per ramet δ was estimated to be 0.63 in year one and 0.79 in year two, and outbred plants produced 2.5 times more ramets than inbred plants in the spring of year two. Inbred plants also suffered more herbivore damage than outbred plants in both fields, suggesting that inbreeding compromises herbivore resistance. Total per genet δ was 0.85 over the two years, indicating that *S. carolinense* is unlikely to become completely self-compatible, and suggesting that plasticity in the SI system is part of a stable mixed-mating system permitting self-fertilization when cross pollen limits seed production.

## Introduction

Self-fertilization is common in plants—it has been estimated that half of all flowering plant species self-pollinate 20% or more of the time [1]—and has pronounced effects on fitness. Because inbreeding reduces heterozygosity, thereby exposing deleterious recessive alleles to selection while decreasing the contribution of over-dominance to fitness, most species show a significant loss of fitness with inbreeding (see reviews by [2]–[4]). Consequently, inbreeding depression, defined as the reduction in fitness of selfed progeny relative to outbred progeny, is a major factor influencing the evolution of plant mating systems: most models of mating system evolution predict a threshold level of inbreeding depression (0.5 in the simplest cases) below which the transmission advantage of selfing favors alleles that increase the selfing rate and above which the reduced fitness of inbred offspring favors alleles that promote outcrossing (e.g., [5]–[8]). Accurate estimates of inbreeding depression are therefore necessary in order to predict the evolutionary trajectory of mating systems.

However, the few studies that have examined the effects of inbreeding at broader spatial and temporal scales (see [2]–[5], [9]) indicate that the magnitude of inbreeding depression is not a fixed property of species or individual populations. Moreover, studies that examined inbreeding depression under both greenhouse and field conditions have generally reported higher levels of inbreeding depression in the field (e.g., [10]–[12]), suggesting that inbred plants may exhibit increased vulnerability to a variety of biotic and abiotic stresses that exhibit considerable natural variation. Despite the apparent need for further investigation in this area, little work to date has examined multi-year estimates of inbreeding depression in perennial plants and no studies have examined the effects of inbreeding on clonal spread in herbaceous perennials.

Insect herbivory is a key biotic stressor in natural plant populations that likely has important interactions with inbreeding (e.g., if inbreeding depression compromises plant resistance or tolerance). Foliar herbivory is ubiquitous in terrestrial ecosystems [13], [14] and has been shown to decrease fitness in a wide variety of species (e.g., [14]–[18]). Given the general loss of vigor typically observed with inbreeding depression, it is reasonable to suspect that inbreeding will increase vulnerability to insect herbivores: inbred plants may spend more time in vulnerable stages of their life cycle; they are likely to have fewer resources to deploy toward defense; and increased homozygosity may expose deleterious recessive alleles for any of the hundreds of genes known to be involved in plant defenses against natural enemies [19]. Recently, researchers have begun to explore the effects of inbreeding on herbivory [18], [20]–[26]. In general, these studies indicate that inbreeding does reduce resistance to herbivores and suggest that the effects of inbreeding on plant-herbivore interactions may have widespread implications for the evolution of breeding systems, herbivore population dynamics, the establishment and transmission of herbivore vectored plant diseases, competitive interactions among plants, and tritrophic plant-herbivore-predator interactions [18], [20]–[24], [26], [27].

In the current study, we directly explored the effects of inbreeding depression on plant fitness under field conditions in the herbaceous perennial weed *Solanum carolinense* (horsenettle), a species that exhibits plasticity in the strength of its self-incompatibility (SI) system [28], [29]. A previous greenhouse study [30] revealed very low levels of inbreeding depression in horsenettle, suggesting strong selective pressure for self-fertility and the possibility that this species might be in transition from SI to self-compatibility. To test this hypothesis under real-world conditions we grew selfed and outcrossed progeny from eight maternal plants in two replicated field plots over two years and measured the effects of inbreeding on herbivore damage, reproductive output, and the number of ramets produced by horizontal (rhizome-like) roots. In the second year, we manipulated insect herbivory in one of our two replicate fields (using chemical pesticides) in order to explicitly measure the influence of herbivory on inbreeding depression.

## Methods

### The study system

*Solanum carolinense* L. (Solanaceae) is an herbaceous perennial weed native to North America that inhabits early successional habitats, waste places, crop fields, and pastures. Once established, it spreads via horizontal roots that extend up to 1 m from the parent stem [31]. The white to violet flowers are visited by pollen-gathering bees, which vibrate the flowers to remove pollen [32]. Most flowers are perfect and functionally hermaphroditic and are born on racemes of 1–12 blossoms; a few, however, (usually located at the tip of the raceme) have reduced pistils and are functionally staminate [23]. The fruit are yellow or orange berries, 1–2.5 cm in diameter, typically containing 60–100 seeds [33], [34]. The reproductive season lasts from early summer until the first frost, when above-ground plant parts die. Below-ground parts over-winter, and new ramets emerge in the spring. Both growth and reproduction are indeterminate.

Horsenettle exhibits a variety of traits that likely play a role in defense against herbivores. Both leaves and stems are covered by spines; leaves are also covered with stellate trichomes; and all parts of the plants contain toxic secondary compounds (e.g., glycoalkaloids), especially the fruits [35], [36], [37]. Despite these defenses, many insects feed on the leaves, fruits, flowers, or roots of horsenettle and several herbivore species have been shown to significantly depress reproductive output (e.g., [33]–[35], [37]–[44]).

*Solanum carolinense* exhibits a typical Solanaceous-type RNase-mediated gametophytic self-incompatibility (GSI) system controlled by the multi-allelic *S*-locus [29], [45]. SI is uncommon in weedy and invasive species (e.g., [46], [47]), presumably because (*i*) disturbed habitats require frequent re-colonization (hence populations are repeatedly founded by one or a few individuals bearing a limited number of *S*-alleles), (*ii*) effective population sizes are small (supporting few *S*-alleles, hence compatible cross pollen may limit fruit and seed production), and (*iii*) habitats are often short-lived (providing limited time for the migration of additional *S*-alleles into populations). Consequently, each time a population is founded, weeds with SI must reproduce despite limited availability of compatible cross pollen or go locally extinct.

Previous studies by our group have investigated the apparent anomaly of SI in horsenettle (i.e., a weed that is a highly successful in early successional habitats despite being self-incompatible) and have found that the SI response in *S. carolinense* is a plastic trait—its strength being affected by the age of the flowers [29] and prior fruit production [30]. Moreover, there are genetic differences among families in their self-fertility [30], [48]. Taken together, these studies demonstrate that, while all horsenettle genotypes are capable of setting self seed when outcross pollen is scarce (older flowers remain unpollinated and/or when few or no outcross fruit are produced on the first 3–5 inflorescences), these effects are more pronounced for plants carrying particular *S*-alleles (plants carrying these alleles set significantly more selfed seed than others) [48]. The importance of this variation in self-fertility on the ability of horsenettle to found and establish new populations depends, to a large extent, on the magnitude of inbreeding depression. We would predict inbreeding depression to be high in horsenettle, as selfing should be fairly uncommon in a species exhibiting an RNase-mediated GSI response. However, a recent greenhouse study revealed that inbreeding depression (δ) for 6 selfed and 6 outcrossed progeny from 16 families was only 0.17 [30].

### Plant Materials

Horsenettle plants were collected from a large natural population located near State College, Pennsylvania. Cuttings were taken from the horizontal roots of 16 plants located at least 5 m apart (in order to decrease the possibility of taking rhizomes from the same genet). These cuttings were brought to the greenhouse, planted in 4-L pots, and allowed to resprout, grow, and flower. After flowering, we cut the stems and moved the pots to a cold room at 4°C to vernalize for 6–8 weeks. Afterward, the potted plants were returned to the greenhouse and allowed to acclimate for 1 week. We then created ramets from each of the 16 plants (genets) by dividing the horizontal root into 5–6 pieces of similar size. Each root cutting was replanted in a 1-gallon pot and allowed to re-sprout and grow. Four of the ramets from each genet were used to produce self (2 ramets) and cross (2 ramets) seeds via hand pollinations. The resulting seeds were germinated and grown in the greenhouse, then used for the greenhouse study of inbreeding depression [30]. The *S*-alleles for each plant were determined using *S*-allele-specific primers in a PCR-based screening protocol (see [30], [48]). After completion of these studies, the plants were cut back and the roots placed into plastic bags and returned to the cold room.

For the present study, we selected 1 self progeny and 1 cross progeny from each of 8 maternal parents. Each of the 16 plants had a unique *S*-allele composition that could serve as a marker for clonal growth under field conditions. A horizontal root from each of these 16 plants was cut into 4 equal-sized (10 cm) pieces; placed into a flat bed with in a peat-based, general-purpose potting soil (Pro-Mix, Premier Horticulture, Quakertown, PA); and allowed to re-sprout in a greenhouse room (16L∶ 8D; day/night temperatures 25/22°C; 65% relative humidity, plants watered lightly each day). After 2 weeks, sprouts were transplanted to 4-L pots (under similar conditions) and watered daily. At the time of transplanting, plants received a fertilizer application (50 ppm 8-45-14 N-P-K, plus micronutrients; Scotts, Marysville, OH) and iron chelate (Sprint 138 at 6%; Becker Underwood, Ames, IA). When the re-sprouted ramets were approximately 6 weeks old (in late May 2008), two randomly selected ramets from each of the 16 genets were transplanted into an abandoned agricultural field at the Entomological Farm of the Pennsylvania State University Agriculture Experiment Station at Rock Springs (planted in barley in the previous year). One ramet of each of the 16 genets was randomly assigned to a location onto the perimeter of a circle that was ∼10 meters in diameter, so that all plants were the same distance from its nearest neighbors (∼2 m). A replicate circle of plants was planted ∼75 meters from the first circle using the other ramet of each genet.

### Year 1

At the end of the growing season (just after the first frost), we harvested and counted the mature fruits from each genet and counted the seeds from a random sample of 5 fruits from each genet (if a plant did not produce 5 fruits we counted the seeds in all of the fruits produced on that plant). Fruits per genet (8 outbred and 8 inbred), mean seeds per fruit per genet, and total seeds produced [mean seeds per fruit×fruits per genet] were analyzed with a mixed effects model ANOVA with replicate, breeding, and family (random) as the main effects.

Plants remained in the field over the winter, and the following June we mapped all ramets that emerged. A sample of leaf tissue was obtained from each ramet, placed in liquid nitrogen, and stored at −80 C until further processing. In order to determine the *S*-genotype of each ramet, we used a modified PCR-based screening protocol, using allele-specific primers [49]. A detailed description of these methods was presented by [48]: briefly, total genomic DNA was extracted from leaf tissue using Plant DNAzol (Invitrogen) and Ribonuclease A (Invitrogen) and re-suspended in 50 µl of DEPC-treated water. Each plant was screened simultaneously for all *S*-alleles present in the population to ensure proper genotype determination and to reduce the possibility of false positive amplification. Selected parental genets comprising all *S*-alleles present in the original population were amplified along with the ramet samples in order to serve as positive controls. The PCR amplification of *S*-alleles was carried out in a 20 µl volume reaction containing 20 ng of DNA, 10× PCR buffer, 0.1 mM of each dNTP, 10 ng of each forward and reverse allele-specific primer, and 1 unit of HotStart Taq DNA polymerase. The reaction was incubated at 95°C for 3 min, followed by 30 cycles of 1 min at 95°C, 1.5 min at 60°C, and 1.5 min at 72°C, and a final extension step of 5 min at at 72°C. For allele *S_18_*, a touchdown protocol was used, with five cycles of 1 min at 95°C, 1.5 min at an initial annealing temperature of 60°C with a 1°C decrease per cycle, and 1.5 min at 72°C, followed by 25 cycles of 1 min at 95°C, 1.5 min at 55°C and 1.5 min at 72°C, and a final extension step of 5 min at 72°C. PCR products were run in a 1% agarose gel and scored for their identity.

### Year 2

In order to determine if leaf herbivory increased inbreeding depression, we randomly chose one of the replicate plots to be hand-sprayed with a carbaryl insecticide (Sevin™) at two-week intervals throughout the growing season. (Our analyses of fruit and seed production in year 1 of this study revealed no effect of replicate on fruit or seed production). We non-destructively estimated leaf damage by herbivores on 15 June, 15 July, and 15 August, using a 0–5 index in which 0 = most leaves with no damage and no leaf with more than 5% of the leaf area removed, and 5 = all leaves damaged and most leaves with >50% of the leaf area removed. Three people, blind with respect to plant family and breeding history, concurrently and independently evaluated damage on each plant. If two or three of the evaluators agreed on the score, we recorded that value. If all three assessments differed (∼5% of cases), we recorded the intermediate score. While estimating leaf damage, we also recorded the types of herbivores that we observed on the plants. After identifying the *S*-alleles for each plant, we assigned each ramet to one of the original 16 genets (one outbred and one inbred plant from each of 8 maternal plants).

To determine the effects of replicate, breeding, and family (random) on the production of new ramets in June of year 2, we used a mixed effects model ANOVA. Because field plot and treatment were confounded in year two, we performed separate mixed effect model ANOVAs on the no spray and sprayed fields to determine the effects of breeding and family (random) on the total number of seeds produced per genet (we combined all of the seeds produced by all ramets of each genet). To determine the effects of herbivore damage on total seeds we performed 4 separate mixed effects model regressions (inbred spray, outbred spray, inbred no spray and outbred no spray) of herbivore damage on total seeds with a random intercept term for each genet. Regressions were performed using the “lme” function in the “nlme” package in the R programming language (R foundation for statistical computing, Vienna). Total seeds were log transformed to approximate normality.

Finally, we calculated inbreeding depression (δ) using the formula δ = (1−seeds _selfed plants_/seeds _outcross plants_) for plants growing in year 1 and 2, and for the two year total seed production per genet. All ANOVAs were performed using Minitab version 16 (Minitab, Inc, State College, PA.).

## Results

During year one, outbred genets made significantly more fruits (30.6±2.6 vs. 14.3±2.6; least square means [LSMeans] ± standard error [SE]), more seeds per fruit (92.4±4.4 vs. 69.3±4.4), and more total seeds per plant (2776±259 vs. 1028±259; δ = 0.63) than inbred genets. Family was marginally insignificant for total fruits per plant and total seeds and marginally significant for seeds per fruit (Table 1). The replicate fields did not differ significantly in fruit or seed production (Table 1).

**Table 1:** Variance analysis for reproductive output in year 1.

*a. Fruits*				
Effect	df	MS	F	P
Replicate	1	36.1	0.34	0.568
Breeding	1	2145.1	19.97	<0.001
Maternal family	7	242	2.25	0.069
Error	22	107.4		

b. *Mean seeds/fruit*				
Effect	df	MS	F	P
Replicate	1	7.5	0.02	0.878
Breeding	1	4043.3	12.98	0.002
Maternal family	7	885.3	2.84	0.029
Error	22	311.5		

c. *Total seeds produced*				
Effect	df	MS	F	P
Replicate	1	767777	0.72	0.407
Breeding	1	24446304	22.78	<0.001
Maternal family	7	2163867	2.02	0.099
Error	22	1073212		

In the spring of year two, 461 ramets emerged on the two replicate fields and each ramet was unambiguously assigned to a genet using *S*-allele specific primers with PCR. Outbred genets produced significantly more ramets than inbred genets (20.9±1.6 vs. 7.9±1.6; LSMeans ± SE). No other factor in the model had a significant effect on ramet production (Table 2).

**Table 2:** Variance analysis for ramet production.

Effect	df	MS	F	P
Replicate	1	140.28	3.44	0.078
Breeding	1	1339.03	63.28	<0.001
Maternal family	7	19.07	0.47	0.847
Replicate×Breeding	1	63.28	1.55	0.226
Error	21	40.75		

**Figure 1:**
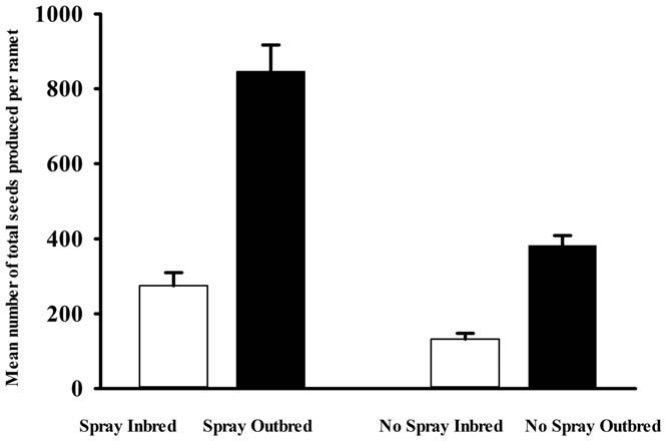
Mean +/− SE for the number of total seeds produced per ramet among inbred and outbred plants on spray (bi-weekly insecticide applications) and no-spray fields during the second growing season.

During the summer of year 2, the outbred genets (all ramets combined for each genet) on both fields produced significantly more total seeds (no spray field = 6708±401 seeds; spray field = 20694±2784 seeds; LSMeans +/− SE) than the inbred genets (no spray field = 993±401 seeds; spray field = 2437±2784 seeds). There were no significant effects of family on seed production (Table 3). The effect of inbreeding on seed production is due to a combination of both greater ramet production on the outbred plants (above) and greater total seed production per outbred ramet (Fig. 1). Inbreeding depression per ramet was greater on the no spray field (δ = 0.79) than on the sprayed field (δ = 0.68). Over both years, the inbred genets on the no spray field produced only 15% of the seeds produced by the outbred plants (δ = 0.85).

**Table 3:** Variance analysis for seed production per genet in year 2.

a) *Spray field*				
Effect	df	MS	F	P
Breeding	1	1333230205	21.51	0.002
Maternal family	7	83828241	1.35	0.35
Error	7	61993408		

*b) No Spray field*				
Effect	df	MS	F	P
Breeding	1	130683765	101.42	<0.001
Maternal family	7	1925976	1.49	0.304
Error	7	61993408		

**Figure 2:**
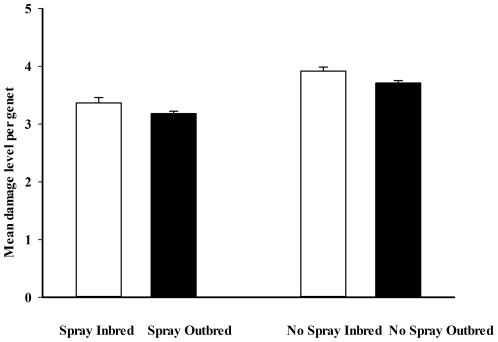
Mean +/− SE for the average amount of herbivore damage on each ramet per genet among inbred and outbred plants on spray (bi-weekly insecticide applications) and no spray fields during the second growing season.

Within both the sprayed field and the no spray field in year 2, the outbred ramets experienced slightly lower levels of herbivory than inbred ramets (Fig. 2). The most abundant herbivores observed while obtaining the estimates of herbivore damage included Flea beetles (*Epitrix spp*), Colorado potato beetles (*Leptinotarsa decem-lineata*), and false Colorado potato beetles (*Leptinotarsa juncta*). Less frequently observed were the tobacco hornworm (*Manduca sexta*), the flower weevil (*Anthonomus spp.*), and larvae of the fruit-infesting moth *Frumenta nundinella*. We also observed several predaceous insects on our plants, including ladybird beetles (*Epilachna spp.*), big-eyed bugs (*Geocoris spp.*) and braconid wasps (*Apanteles spp.*). Our regression analyses revealed no significant relationship between our estimates of herbivore damage and reproductive output for any of the four field-breeding combinations: inbred plants on the non-sprayed field, outbred plants on the non-sprayed field, inbred plants on the sprayed field or outbred plants on the sprayed field (all p>0.18).

## Discussion

### Inbreeding depression under field conditions

This study examined the effects of inbreeding on fruit and seed production of horsenettle (*Solanum carolinense*) over two years under field conditions—to our knowledge no previous studies have examined inbreeding depression in a clonally spreading herbaceous perennial over multiple years. Unfortunately, our desire to track ramet production across years (i.e., to estimate the per-genet inbreeding depression in this clonal herbaceous perennial) also necessitated tradeoffs in sample size including the number of inbred and outbred progeny per family, number of families, and number of replicate plots.

In the first growing season inbred plants produced only 37% as many seeds as outbred plants. The resulting estimate of inbreeding depression (δ = 0.63) is much higher than that previously reported from a greenhouse study that employed a larger set of horsenettle genets including the 16 used in the present study (δ = 0.17) [30], [50]. Consequently, this study joins a growing list of reports that measurements of inbreeding depression under benign conditions (e.g., those obtained from greenhouse studies) tend to underestimate the intensity of inbreeding depression occurring under more stressful field conditions (e.g., [6]–[8]).

We furthermore found that outbred genets produced>2.5× as many ramets as inbred genets in the spring of year two, and that inbreeding depression increased both per ramet and per genet from year 1 to year 2. Only a few previous studies have examined inbreeding depression in the same population over two or more years under field conditions (e.g., [5], [51], [52]). These studies also found annual variation in the magnitude of inbreeding depression—presumably due to environmental variation in nutrient availability, rainfall, and various biotic stresses that can alter the resources plants are able to allocate to reproduction. Thus, the magnitude of inbreeding depression appears not to be an intrinsic property of particular populations (or families) but rather a context-dependent measure of the enhanced sensitivity of inbred plants to the challenges posed by variable real-world environments [52].

Previous studies that have expressly examined the interaction of environmental stressors with inbreeding have reported that inbreeding depression increases with competition (e.g., [52]–[55]), drought (e.g., [56]), and nutrient stress (e.g., [5], [57]). In the current study, we observed that inbred plants had more herbivore damage than the outbred plants under field conditions. Consistent with previous studies of horsenettle (e.g., [34], [38], [43]), we found that the plants in our fields were attacked by an array of herbivores that feed predominantly on Solanaceous species. In another recent study, we found that tobacco hornworm larvae (*Manduca sexta*) preferred to feed on horsenettle leaf disks from inbred versus outbred plants and also exhibited higher levels of total leaf consumption and higher relative growth rates on the inbred plants [58]. Those results, together with the current data, contribute to the growing body of evidence that inbreeding alters resistance to herbivores and often improves plant quality as a food source for herbivores [18], [20]–[26].

We also found that application (every other week) of an insecticide on one of our two replicate fields yielded small decreases in overall levels of herbivory that coincided with similar changes in estimates of inbreeding depression per ramet (from 0.79 to 0.68 in year 2). It must be noted that the lack of treatment replication in this experiment means that treatment and field location were confounded, though the absence of significant field effects on reproductive output or ramet production in year one mitigates this concern to some extent. Thus, even though there were no significant differences in seed production between the two fields during the first year of this study, we cannot unambiguously attribute the decrease in per ramet inbreeding depression to the insecticide treatment (and consequent reduction in herbivory). But, regardless of the underlying causes of variation in per-ramet inbreeding depression between the two fields in year 2 (the spray treatment or other environmental differences between the fields), this study produced three estimates of δ in two years, ranging from 0.63–0.79. Although per-ramet estimates of inbreeding depression could increase or decrease from year to year in the same population as environmental conditions vary, the dramatic difference in ramet production that we found in the inbred and outbred horsenettle plants suggests that per genet estimates of inbreeding depression are likely to be amplified across subsequent years of clonal spread.

Few studies have examined the specific mechanisms underlying the effects of inbreeding on herbivore preference and performance, which are known to be influenced by factors such as variation in plant nutritional quality, constitutive and induced chemical defenses, and the induced production and release of volatile compounds that can be attractive to herbivores' natural enemies (e.g., [59]–[64]). As with the vast majority of studies of inbreeding depression (e.g., [3]), inbred plants in our study exhibited slower growth (i.e., they produced far fewer ramets) and reduced reproductive output relative to outbred plants, suggesting they are likely to linger in vulnerable stages of development and have fewer resources to devote to chemical defenses and volatile signaling. In another recent study, we documented broad sense heritable variation for whole plant volatile production by horsenettle and found that outbred plants produce significantly greater total volatiles than inbred plants under field conditions (natural herbivory), suggesting that inbreeding may indeed impact volatile-mediated interactions between herbivores and their natural enemies [65].

Although inbreeding reduced resistance to herbivores, our regression analyses revealed no relationship between the amount of herbivore damage and reproductive output per ramet. This is somewhat surprising because previous studies have shown that herbivory reduces reproductive output in horsenettle (e.g., [35], [39], [41]); moreover, our analyses show that plants in the insecticide-sprayed field had lower levels of herbivory and greater reproductive output than plants in the unsprayed field and that inbreeding depression was greater in the no-spray (high-herbivory) field. It is possible that our non-destructive field estimates of herbivore damage were simply too crude to detect the effects of herbivory on plant reproduction. Furthermore, our estimates did not differentiate among damage caused by different types of herbivores. Each of the common herbivores that we observed caused different patterns of damage, and the amount of damage caused by each type of herbivore varied over time. Several recent studies have shown that tolerance to herbivory can vary with the pattern of damage and with ontogenetic stage (e.g., [24], [66]–[68]).

### Evolution of the horsenettle breeding system

Our results have profound implications for the evolution of the breeding system in *Solanum carolinense*. Previous work has demonstrated plasticity in the SI response of horsenettle [28], [29]. Horsenettle flowers become more self-fertile with age and when few or no cross-pollinated fruits are developing on a plant (i.e., when cross pollen limits seed production). Moreover, we have shown that plasticity in SI is enhanced in the presence of the “leaky” *S9* allele [48]. It is generally thought that most mutations that enhance self-fertility are eliminated by genetic drift or by purifying selection effected by inbreeding depression (e.g., [7], [8]). On the other hand, mutations that enhance self-fertility in a population exhibiting pollen-limited seed set (e.g., [69]) and/or low to intermediate levels of inbreeding depression [8], [67] may become fixed, resulting in the loss of SI. Indeed, the transition from obligate outcrossing via SI to self-compatibility is among the most common evolutionary pathways in flowering plant genera [70], [71].

Traditionally, populations of SI species with *S*-allele polymorphisms for enhanced self-fertility (or genes that modify the strength of SI) have been viewed as either temporarily harboring some self-fertility alleles or in transition to self-compatibility. However, there has recently been considerable theoretical interest in the possibility that polymorphisms for enhanced self-fertility could also be the product of selection for a stable mixed-mating system (e.g., [70], [72]–[75]). These theoretical studies reveal that the broadest conditions for the stability of such polymorphisms in natural populations occur when (1) there are low *S*-allele numbers and/or high rates of pollen limitation in the population; (2) there are high levels of inbreeding depression and/or *S*-linked/sheltered load (*sensu* [8]); (3) the self-fertility enhancing alleles promote delayed self-fertilization (and therefore do not decrease opportunities for cross-fertilization); and (4) the self-fertility enhancing alleles confer only a small increase in the rate of self-fertilization. Our previous findings [28], [29], [48], [50] suggest that increases in the rate of self-fertilization only occur in populations in which seed production is limited by cross pollen, and that self-fertilization is unlikely to limit the opportunities for cross-fertilization. Moreover, because horsenettle is a weed prone to repeated bouts of colonization and extinction, the conditions that favor self-fertility are likely to occur commonly (i.e., few *S* alleles in a population and reproduction that is limited by the availability of cross pollen).

The data presented here reveal that inbreeding depression in horsenettle under field conditions is significant in a given year and that total inbreeding depression over the lifetime of a genet is likely to be severe. Consequently, *S. carolinense* is likely not in transition from SI to self-compatibility. Rather, the plasticity in the SI system and the presence of the leaky *S9* allele—which is a common and widespread in the Eastern United States [45], [48]—may be part of a stable mixed mating system that permits the plants to self-fertilize when cross pollen limits seed production and/or when few S-alleles are present in the population.

In conclusion, this study clearly demonstrates that (1) estimates of inbreeding depression for *S. carolinense* are far greater under field conditions than under greenhouse conditions; (2) inbreeding reduces vegetative growth via clonal spread; (3) estimates of inbreeding depression per ramet can vary with year and local environmental conditions (e.g., the intensity of herbivory); (4) inbreeding depression per genet is severe and likely to increase over time due to the reduced clonal spread of inbred ramets; and (5) inbred plants suffer more herbivory than outbred plants. These results, taken together with our previous studies of the plasticity in the SI system of horsenettle and viewed in light of insights from recent theoretical investigations, suggest that plasticity in the SI system of *S. carolinense* is part of a stable mixed mating system that favors outcrossing except where cross pollen severely limits seed production (as might occur when founding new populations). Future studies should focus on documenting the mechanisms underlying the increased levels of herbivory observed on inbred plants and the rates of selfing in small populations with few *S*-alleles and larger, established populations with many *S*-alleles.
